# All-Trans Retinoic Acid-Induced Ototoxicity during Chemotherapy in Pediatric Acute Promyelocytic Leukemia

**DOI:** 10.3390/children8010027

**Published:** 2021-01-06

**Authors:** Jong Hyeon Lee, Jong Ho Lee, Jae Min Lee

**Affiliations:** 1 Department of Pediatrics, Yeungnam University Medical Center, Daegu 42415, Korea; leejh931017@gmail.com; 2 Department of Laboratory Medicine, College of Medicine, Yeungnam University, Daegu 42415, Korea; leejongho@ynu.ac.kr; 3 Department of Pediatrics, College of Medicine, Yeungnam University, Daegu 42415, Korea

**Keywords:** acute promyelocytic leukemia, all-trans retinoic acid, tinnitus, hearing loss

## Abstract

All-trans retinoic acid (ATRA) is known to induce complete remission of acute promyelocytic leukemia (APL) and its use has significantly improved the cure rate of APL. However, ATRA also causes side effects such as differentiation syndrome or intracranial hypertension. In our case, the patient was diagnosed with APL and developed hearing loss thrice while being treated with ATRA. Therefore, we reduced the dose of ATRA instead of stopping it altogether and administered dexamethasone to the patient. A hearing test performed thereafter revealed recovery of hearing. No recurrence of hearing loss occurred after prednisolone and ATRA were combined in the maintenance phase. In conclusion, ATRA-associated hearing loss is reversible, and it is not necessary to stop ATRA. We recommend completion of a randomized clinical trial using dexamethasone in combination with ATRA to prevent hearing loss caused by ATRA.

## 1. Introduction

Acute promyelocytic leukemia (APL) is a type of acute myeloid leukemia (AML). The cure rate of APL has improved remarkably since the introduction of all-trans retinoic acid (ATRA) as a treatment [1]. However, ATRA use is typically and commonly associated with differentiation syndrome (DS), which is caused by an inflammatory response mediated by an ATRA-induced increase in promyelocyte maturation and cytokine expression in APL patients. DS is known to occur in 20–30% of patients undergoing ATRA treatment [2]. However, hearing impairment during ATRA treatment is rare [3]. We report a rare case of a 13-year-old patient with APL who developed hearing impairment during ATRA treatment.

## 2. Case Report

**Figure 1:**
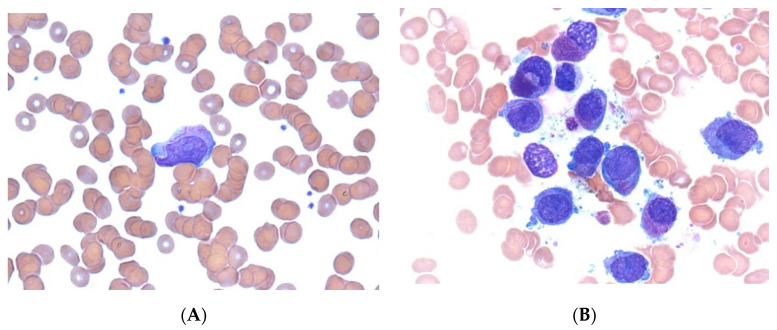
Acute promyelocytic leukemia. Peripheral blood smear (**A**) and bone marrow aspirate smear (**B**) showing abnormal promyelocytes. Some of the cells show intense azurophilic granulation and bundles of numerous Auer rods (Wright’s stain, ×1000).

This report was approved by the institutional review board (IRB) of Yeungnam University Hospital (IRB No: YUMC 2020-07-037). Written informed consent was obtained from the patient and guardians for the publication of this case report and the accompanying images. The patient was a 13-year-old boy who presented with fever, leukopenia, and thrombocytopenia. Bone marrow aspiration and examination revealed hypercellular marrow with 89% of the promyelocytes being abnormal, and PCR showed promyelocytic leukemia (PML)/retinoic acid receptor, alpha (RARα) positivity. Based on these findings, a diagnosis of APL was made (Figure 1).

**Figure 2:**
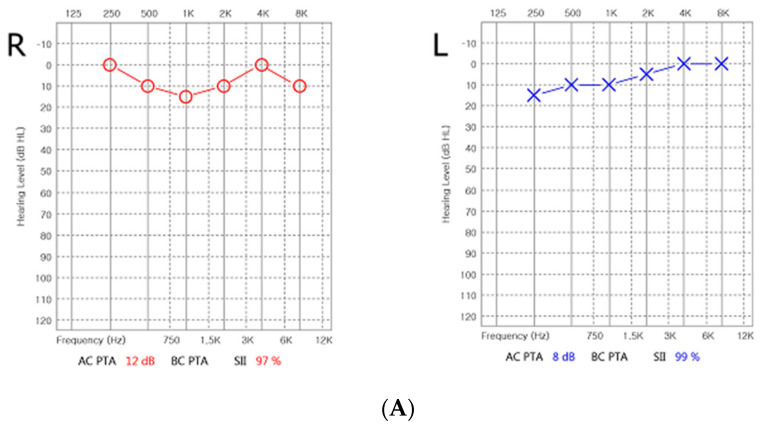

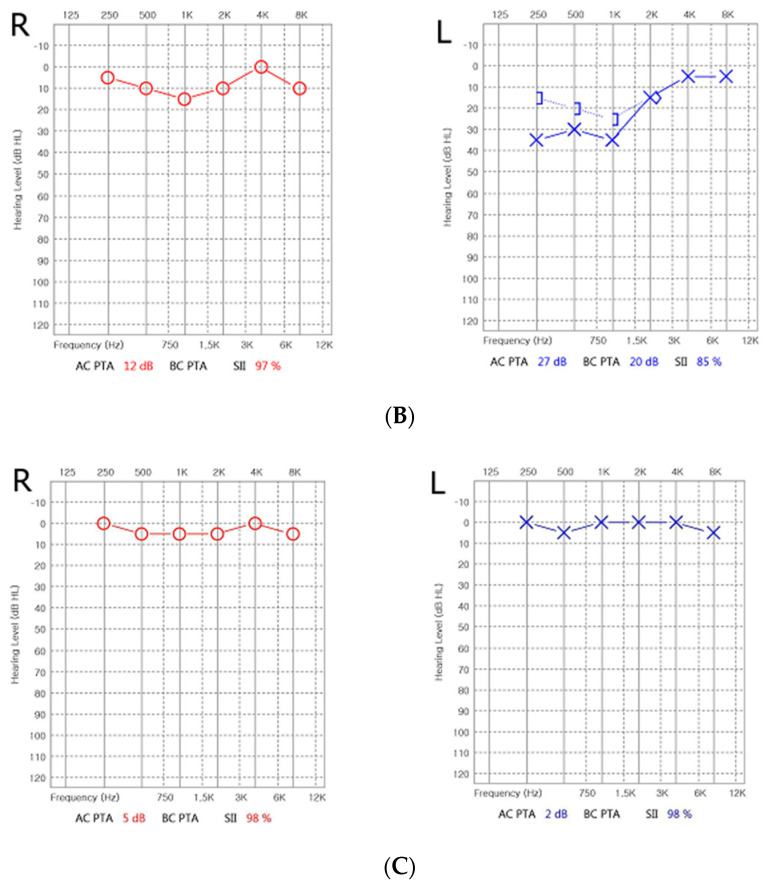
Serial audiometry of patients. (**A**) Pure tone audiometry (PTA) did not reveal any abnormality at first event of tinnitus. (**B**) Two weeks thereafter, in the first consolidation period, ATRA was used and the patient developed headache and tinnitus in both ears. Severe hearing loss at 250 Hz, 500 Hz, and 1000 Hz on the left side at 2 weeks later. (**C**) One month later, PTA showed improvement after treatment of dexamethasone.

Treatment with ATRA (45 mg/m^2^/day) was administered immediately after APL was suspected; chemotherapy was started according to the modified AIDA-2000 protocol. Dexamethasone was added for prophylaxis of DS. Brain MRI was performed because the patient developed headache 2 days after the start of ATRA treatment; it revealed no bleeding or mass. On physical examination, papilledema was also not observed. After the induction phase, the patient suddenly developed tinnitus of the left ear. Pure tone audiometry (PTA), speech audiometry, impedance audiometry, and tinnitogram did not reveal any abnormality (Figure 2A). Considering the possibility of sensorineural hearing loss (SNHL) associated with ATRA, ATRA was discontinued and dexamethasone 10 mg/day was started. Tinnitus improved immediately and a low-dose of ATRA (25 mg/m^2^/day) was started 1 week later.

Two weeks thereafter, in the first consolidation period, ATRA was used and the patient developed headache and tinnitus in both ears. Therefore, the hearing tests were repeated; hearing loss was detected in the left ear (Figure 2B). Compared to the previous tests, the repeated tests revealed significant hearing loss at 250 Hz, 500 Hz, and 1000 Hz. Hence, ATRA was discontinued again, and dexamethasone 10 mg/day was administered. One week later, tinnitus improved, and hearing tests performed a month later showed remarkable improvement compared to the findings of the previous tests (Figure 2C).

Tinnitus recurred when ATRA was restarted in the third consolidation period; therefore, ATRA was withdrawn and dexamethasone was restarted. Consequently, tinnitus resolved in the same manner as before. A bone marrow aspiration test performed after the consolidation period showed normal findings. In the maintenance period, prednisolone (40 mg/m^2^) was used to prevent ATRA-induced ototoxicity when using ATRA (25 mg/m^2^). Currently, the third maintenance phase is underway and ototoxicities such as tinnitus have not recurred.

## 3. Discussion

APL, a subtype of AML, is caused by the fusion of the *RARα* gene on chromosome 17 and the *PML* gene on chromosome 15. This recruits transcription co-repressors, which induce transcription repression and production of abnormal promyelocytes. These leukemic cells have been reported to be relatively sensitive to anthracycline and cytosine arabinoside combination chemotherapy, which yielded a complete remission (CR) rate of up to 50% in patients with APL [4]. Despite the high sensitivity to chemotherapy, the mortality rate among patients with APL is high due to early coagulopathy. With the advent of ATRA in 1985, a new era began in APL treatment [5]. ATRA induces the dissociation of the transcription co-repressors from the gene and activates transcription. Its initial biologic effects are characterized by the differentiation of malignant cells into phenotypically mature myeloid cells [6]. Hence, the cure rate of APL has improved significantly since the use of ATRA, resulting in more than 85% of patients achieving CR [7].

ATRA is now recognized as the major component of treatment for APL patients; however, it also has side effects. The most commonly reported side effect of ATRA use is DS. However, hearing impairment during ATRA treatment has been rarely reported. Fujiki et al. reported the case of a 12-year-old female patient with APL who developed hearing impairment during ATRA treatment [3]. This patient complained of headache accompanied by nausea during multidrug chemotherapy with ATRA. After dexamethasone medication, the symptoms improved immediately; however, nausea and headache recurred 1 week later along with low tone tinnitus of the right ear. PTA revealed right SNHL at low frequencies. All symptoms improved 10 days after the injection of dexamethasone.

Libien et al. insist that the cause of hearing loss is ATRA-related hearing impairment associated with increased intracranial pressure (IICP) [8]. Cerebrospinal fluid (CSF) contains a complex consisting of retinol, retinol-binding protein, and transthyretin, which are precursors of ATRA. Increased ATRA causes gene expression of a molecule by binding to specific nuclear retinoic acid receptors (RARα, β, and γ) and inhibits CSF absorption in the arachnoid granulation cap cells, ependymal or glial cells, and even in lymphatic pathways, thereby increasing ICP [9]. The authors argue that the imbalance in the internal canal and external lymph caused by IICP is related to hearing impairments in ATRA-treated patients.

In our case, the patient developed hearing impairment after headache during the induction phase. PTA showed significantly reduced hearing in the left ear at 250, 500, and 1000 Hz; this finding was similar to that in the previously mentioned case in which the patient’s hearing improved after treatment with dexamethasone [3]. However, hearing impairment occurred two more times, and no longer occurred after prophylactic use of prednisolone (45 mg/m^2^) during the maintenance period. The cause of hearing impairment was thought to be an imbalance between internal and external lymph due to IICP, as Fujiki et al. insisted. Since ICP was not measured through lumbar puncture, it is difficult to describe a clear causal relationship between hearing impairment and IICP. Nevertheless, the possibility is high because the findings suggested IICP, for example headache before the development of hearing impairment.

DS could also cause hearing loss. DS is characterized by clinical symptoms, such as fever, dyspnea, weight gain, hypotension, or acute kidney failure. Montesinos et al. reported that 24.8% of patients with APL treated using ATRA developed DS [10]. DS induces an inflammatory response by inducing promyelocyte maturation and cytokine expression. The resulting inflammation can cause endothelial damage in various systems. This inflammation could cause vestibulocochlear neuritis, which results in hearing loss. Thus, the possibility of hearing impairment due to DS may be considered; however, it should be noted that the symptoms would mainly manifest at the beginning of ATRA treatment. Hearing impairment should not be present when the tumor burden decreases over time. In the case of our patient, the tumor burden decreased after the induction and consolidation periods, and hearing impairment recurred even after the bone marrow study indicated CR. Therefore, there is a low likelihood of DS causing hearing impairment.

Recent experts recommend ATRA 45 mg/m^2^ in adults, but a reduced dose of 25 mg/m^2^ in children, ATRA is often recommended for pediatric patients because of their greater risk of developing intracranial hypertension [11].

ATRA-induced SNHL is a rare but possible side effect. If SNHL develops in a patient treated with ATRA, it is necessary to evaluate hearing impairment by conducting PTA and rule out intracranial hemorrhage by performing CT or MRI. Steroids can be used to manage inflammation, and could be used as prophylaxis if SNHL recurs.

## Data Availability

Raw data is not made publicly available due to patient confidentiality.

## References

[1] Stahl M., Tallman M.S. (2019). Acute promyelocytic leukemia (APL): Remaining challenges towards a cure for all. Leuk. Lymphoma.

[2] Stahl M., Tallman M.S. (2019). Differentiation syndrome in acute promyelocytic leukaemia. Br. J. Haematol..

[3] Fujiki T., Nishimura R., Ikawa Y., Noguchi K., Mase S., Kuroda R., Araki R., Maeba H., Yachie A. (2018). Hearing impairment accompanied with low-tone tinnitus during all trans retinoic acid containing chemotherapy. Pediatr. Blood Cancer.

[4] Bernard J., Weil M., Boiron M., Jacquillat C., Flandrin G., Gemon M.F. (1973). Acute promyelocytic leukemia: Results of treatment by daunorubicin. Blood.

[5] Wang Z.-Y., Chen Z. (2008). Acute promyelocytic leukemia: From highly fatal to highly curable. Blood.

[6] Breitman T.R., Selonick S.E., Collins S.J. (1980). Induction of differentiation of the human promyelocytic leukemia cell line (HL-60) by retinoic acid. Proc. Natl. Acad. Sci. USA.

[7] Tomita A., Kiyoi H., Naoe T. (2013). Mechanisms of action and resistance to all-trans retinoic acid (ATRA) and arsenic trioxide (As_2_O_3_) in acute promyelocytic leukemia. Int. J. Hematol..

[8] Libien J., Kupersmith M.J., Blaner W., McDermott M.P., Gao S., Liu Y., Corbett J., Wall M. (2017). Role of vitamin A metabolism in IIH: Results from the idiopathic intracranial hypertension treatment trial. J. Neurol. Sci..

[9] Brinker T., Stopa E., Morrison J., Klinge P. (2014). A new look at cerebrospinal fluid circulation. Fluids Barriers CNS.

[10] Montesinos P., Bergua J.M., Vellenga E., Rayón C., Parody R., de la Serna J., León A., Esteve J., Milone G., Debén G. (2009). Differentiation syndrome in patients with acute promyelocytic leukemia treated with all-trans retinoic acid and anthracycline chemotherapy: Characteristics, outcome, and prognostic factors. Blood.

[11] Sanz M.A., Fenaux P., Tallman M.S., Estey E.H., Löwenberg B., Naoe T., Lengfelder E., Döhner H., Burnett A.K., Chen S.-J. (2019). Management of acute promyelocytic leukemia: Updated recommendations from an expert panel of the European LeukemiaNet. Blood.

